# Computerized tomographic angiography in patients having eSVS Mesh^®^ supported coronary saphenous vein grafts: intermediate term results

**DOI:** 10.1186/1749-8090-9-126

**Published:** 2014-08-13

**Authors:** Uwe Klima, Abdalla A Elsebaey, Mohamed R Gantri, Jochen Bongardt, Gary Miller, Robert W Emery

**Affiliations:** American Hospital Dubai, Dubai, United Arab Emirates; St. Joseph’s Hospital, Houston, USA

**Keywords:** Vein graft patency, External vein support

## Abstract

**Background:**

The Saphenous Vein (SVG) is used in over 80% of coronary artery bypass procedures (CABG) and SVG patency is the Achilles heel of CABG. To address this issue, the eSVS Mesh®, an external Nitinol knitted mesh, fitted like a sleeve over the vein graft preventing over expansion in the high pressure arterial system, has been introduced to improve disease management. Patency data is limited. The objective of this retrospective study is to report patency rates (>3 months) in patients having external mesh support as part of CABG.

**Methods:**

From October 25, 2010 through February 13, 2012, 21 patients had external mesh support of SVG grafts in addition to internal thoracic artery grafting to the Anterior Descending artery. Patients were invited to return for patency evaluation using Computerized Tomographic angiography (CTA) an average of 7.2 months post-operative (R = 3-14 months).

**Results:**

21 male patients (age 57 +/- 9 years) underwent on-pump surgery. The eSVS Mesh was successfully placed on all SVGs. All grafts were determined patent intra-operative by transit time Doppler measurement and there were no operative revisions. There was no operative mortality. 12 of the 21 contacted patients returned for CTA, 8 non-returning patients contacted were alive and asymptomatic but refused to return due to travel restrictions or cost. One patient was lost to follow up. 11 returning patients underwent CTA. One patient was excluded (asymptomatic) due to elevated creatinine. Of the 23 anastomoses in 11 patients (Average: 2.09 grafts/patient) using SVG available for examination, 21 were patent (92%).

**Conclusions:**

In this retrospective non-randomized experience, the external mesh supported grafts displayed excellent intermediate patency.

**Electronic supplementary material:**

The online version of this article (doi:10.1186/1749-8090-9-126) contains supplementary material, which is available to authorized users.

## Background

Coronary Artery Bypass Grafting (CABG) has been shown to prolong life expectancy in patients with left main, triple vessel and/or single vessel disease with stenosis of the proximal left anterior descending artery and in those with triple vessel disease and depressed left ventricular function as well as diabetic patients [1–6]. Complete revascularization has long been a hallmark advantage of surgical coronary revascularization [7]. In spite of the fact that the use of bilateral internal thoracic arteries have a proven survival and decreased major adverse cardiac and cerebral events rate at out to thirty years post-CABG, the use of the saphenous vein is necessary in over 80% of surgical cases to complete revascularization [1, 8, 9]. The Achilles heel with the saphenous vein over the long term is its lower patency rate as compared to arterial grafting [10, 11]. SVG loss can be up to 50% in 10 years and is related to placing the saphenous vein, normally subject to lower venous pressures into the arterial system.

With the lack of muscular walls seen in arteries, when placed under arterial pressure, the vein dilates. Because of the acute impact of arterial pressure, damage to the endothelium can occur, resulting in the thrombus formation of and subsequent lipid deposition. Smooth muscle cells proliferate in the medial layer of the vein wall and migrate to the intimal surface a process known as neointimal hyperplasia. The resulting build up of smooth muscle cells secrete inflammatory and growth factors leading to graft stenosis and ultimate failure [12]. Further, compared to coronary arteries, saphenous veins have a larger luminal diameter resulting in a vein graft to coronary artery mismatch creating blood flow abnormalities including eddy currents and stasis. Experimentally, downsizing the SVG with an external support correlates with more arterial like healing and these grafts develop less intimal hyperplasia [13, 14]. With the concomitant resulting changes noted above, the major cause of graft failure after one month is the development of graft atherosclerotic disease and thrombosis. To address these changes the eSVS Mesh (Kips Bay Medical Inc, Minneapolis, MN) was developed.

The eSVS Mesh is an external support device that is used to downsize the vein graft of up to 35%, making a more appropriate vein to coronary artery match improving the pattern of blood flow. Also, importantly, because of the constrictive external support, expansion of the vein graft cannot occur to more than 8% of its diameter, obviating endothelial injury with the exposure to acute arterial pressure. Animal experimentation documented improved graft healing in arterial like fashion with less neointimal formation in supported grafts as opposed to unsupported grafts [13–15]. A prospective randomized “first in man” feasibility trial was conducted and at the end of one year supported and unsupported grafts had equivalent patency (FDA submission). CE Mark approval was obtained in Europe. Post market studies are continuing. The object of the current study was to obtain further data as to the intermediate term (>3 month) patency in patients receiving the eSVS Mesh external support graft as a part of their coronary revascularization procedure.

## Methods

The eSVS Mesh is an external support device available in three sizes which accommodates veins 3.5 to 7.0 mm when gently distended. The mesh is a highly flexible Nitinol knitted, kink resistant prosthesis into which the vein graft is inserted.

Included in the eSVS Mesh packet is a sizing tool to measure the gently distended diameter of the vein and a double wall thickness. Both of these measurements are critical in choosing the appropriate size and application of the eSVS Mesh. Veins larger than 7.0 mm and smaller than 3.6 mm and/or are too thick to fit the double wall measure should not be supported [16]. Due to radial constriction of the SVG by the eSVS Mesh, larger veins can be supported in smaller eSVS Mesh devices which are available in 3.5, 4.0 and 4.5 mm sizes.

The current study was conducted after ethics committee approval of the Medical Advisory Board and Board of Directors at the American Hospital Dubai (AHD) and individual patient written informed consent obtained. The study was conducted in accordance with the ethical principles and Declaration of Helsinki.

All patients having the eSVS Mesh as a portion of their coronary bypass grafting from October 1, 2010 through February 28, 2012 were contacted after three months or more following their procedure and were asked to return for CTA the results of which were read at AHD. The study is retrospective.

Twenty-one patients, all male, had the eSVS Mesh used on all the saphenous veins as part of their CABG over the study time frame. SVGs were harvested using an open bridging technique and all procedures were conducted on cardiopulmonary bypass with cardioplegic arrest. The eSVS Mesh was able to be deployed in all cases and there were no device related complications. No sequential grafting was performed. The grafts were determined to be patent intra-operatively using transit-time Doppler flow measurement and no revisions necessary. All patients were placed on aspirin and a statin, the dosage depending on their cholesterol level, post-operative. There was no operative mortality, re-exploration for bleeding or sternal wound infection.

Successful contact was accomplished in 20 of 21 patients (95%): All were alive and asymptomatic. One patient was unable to be contacted. Eight patients contacted by phone refused to participate in CT angiography, seven due to travel related restrictions or cost and one due to hospital administrative conflict. Those having travel restrictions were from other countries in the Middle East. Twelve patients (57%) returned for a follow-up. The eSVS Mesh was used to support all of the 26 vein grafts in these patients.

Numbers are expressed as mean +/- standard deviation.

## Results

The mean age of the 12 patients returning for CTA, the crux of this report, was 57 +/- 8.8 years and all were male; the demographics of these patients is shown in Table 1. Patients had 1 to 3 grafts all supported by eSVS Mesh with a total of 26 saphenous vein grafts in addition to internal mammary artery grafting to the anterior descending coronary artery. The eSVS Mesh qualifying sizes are shown in Table 2. There was an average of 2.2 SVG per returning patient. Procedures concomitant to coronary bypass surgery were performed in 3 patients: one had thromboendarterectomy of the distal right coronary artery, one repair of an ASD and one aortic root aneurysm reconstruction. The time interval from the operative procedure to CTA was 7.2 +/- 3.7 (range = 3 to 14) months.

**Table 1:** **Demographics on patients have eSVS Mesh supported saphenous vein graft, returning for angiography 7.2 months post-operative coronary artery bypass**

Baseline history	Mean or % of subject
	(N = 12)
Previous MI	50% (6)
Hypertension	75% (9)
Dyslipidemia	67% (8)
Diabetes	33% (4)
Previous CABG	8% (1)
Previous CVA	8% (1)
HX Tobacco use	
Current	8% (1)
Former	50% (6)
Never	42% (5)
CAD in 1° Relative	83% (10)
NYHA Class	
No/Unknown	42% (5)
Class I	8% (1)
Class II	17% (2)
Class III	25% (3)
Class IV	8% (1)

**Table 2:** **Sizes of eSVS Mesh used to support saphenous vein grafts in patients returning for angiography 7.2 months post-operative coronary artery bypass**

Qualifying device size	Number SVGs/eSVS Mesh (n = 26 grafts)
3.5	27% (7)
4.0	50% (13)
4.5	23% (6)

**Figure 1:**
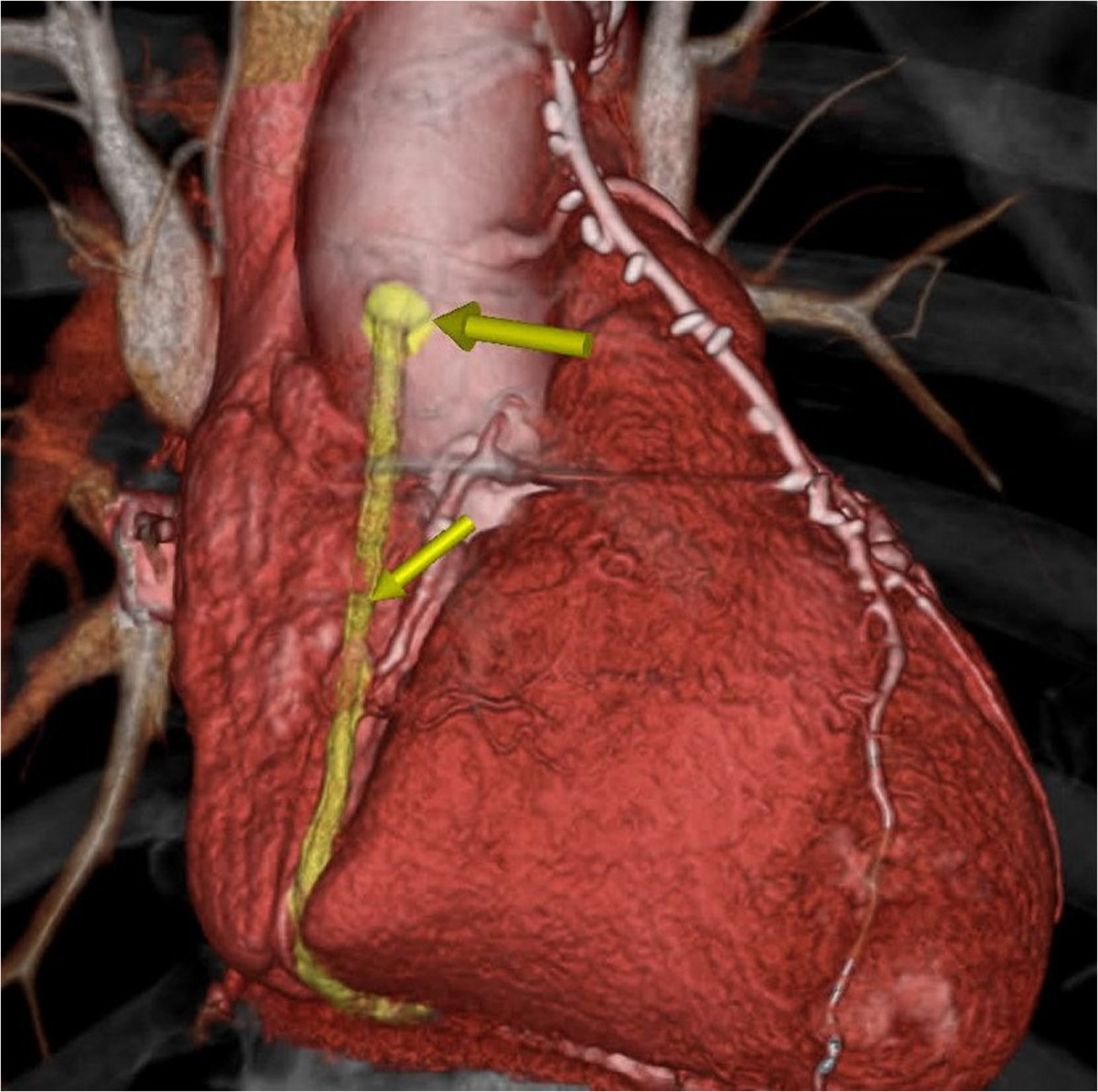
**Representative CT angiogram in a patient multiple patent supported saphenous vein grafts.**

**Figure 2:**
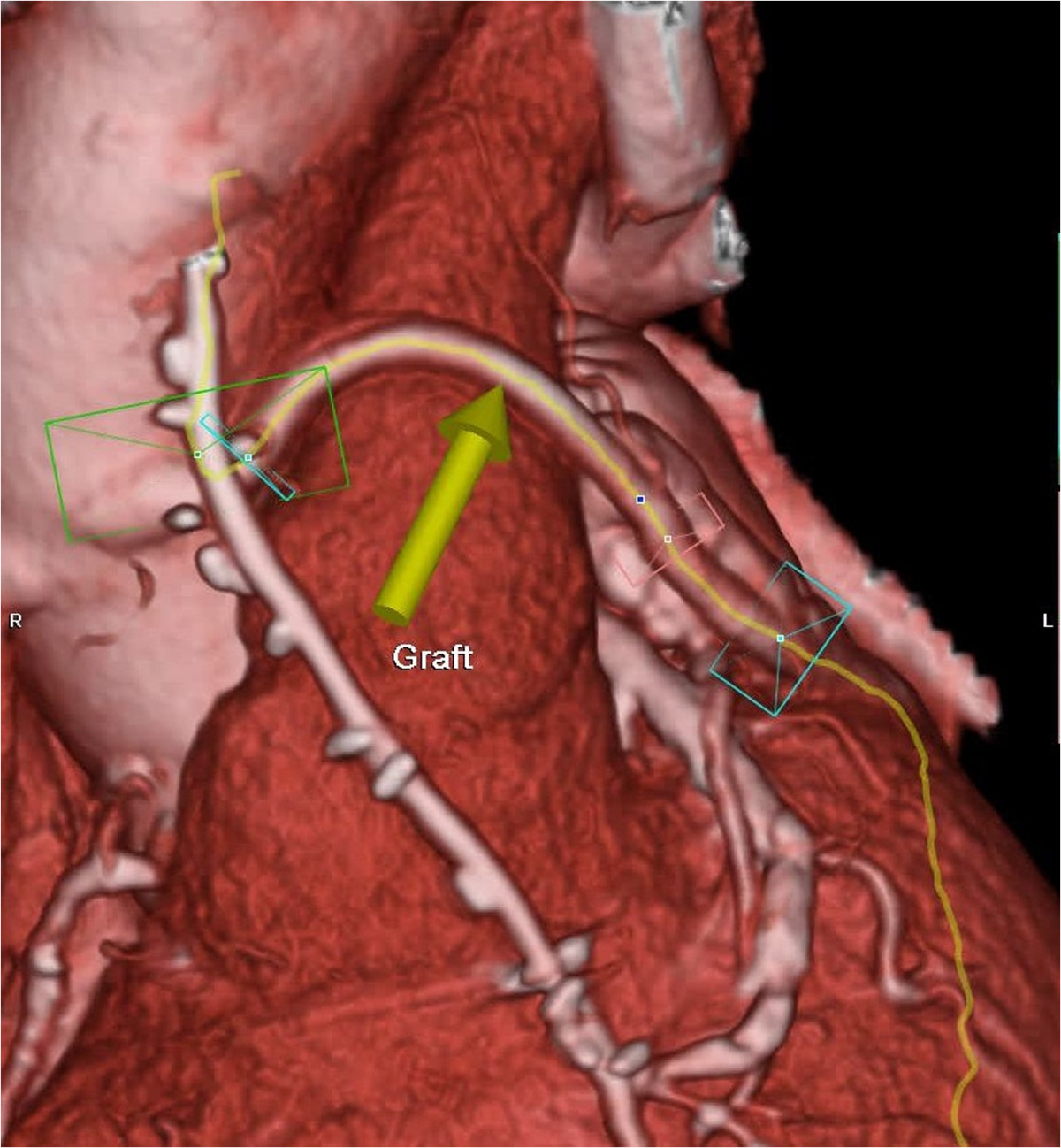
**Representative CT angiogram in a patient with an occluded supported saphenous vein graft.**

In the 12 patients, the eSVS Mesh supported graft was placed in the right coronary position in 9 of 12 patients (75%) and at least one supported graft was placed to the circumflex system in 92% of patients (11 of 12). Three patients had supported grafts placed to a diagonal branch of the anterior descending system. All vein grafts had only one distal anastomosis thus 35% of grafts were placed to the right system (9 of 26) and 65% in the left system (17 of 26). Patients were discharged at mean of 9.1 +/-3.9 days post-operative.Of the 12 returning patients, 11 underwent CTA in which 23 Mesh supported SVG’s (2.09 grafts/patient) were assessed. One patient having 3 supported grafts was enrolled but was unable to have CT angiography due to elevated Creatinine. He is alive and asymptomatic. CTA (N = 11) revealed that 21 of the 23 grafts were patent (patency rate = 92%). There were 2 occluded grafts, one to the right coronary artery and one to the circumflex artery. These CTAs were completed at 6 and 8 month post-operative. A representative CT scan in a patient with multiple patent supported grafts is shown in Figure 1, and an example of a patient with an occluded graft is shown in Figure 2. One patient had a stenosis of 60-70% noted in the mid-portion of a graft. The LIMA graft to the LAD was patent in all cases.

## Discussion

Use of the saphenous vein in coronary bypass surgery is necessary in over 80% of cases to assure completion of the bypass graft procedure [7]. The use of the bilateral internal mammary artery is an important aspect of coronary bypass grafting to compete with stent graft placement; however, saphenous vein grafting is still warranted in the majority of cases [1, 6, 8, 11]. Early saphenous vein patency (<1 week) is substantively improved by anti-platelet therapy delivered 1–6 hours post-operative and combined with cholesterol lowering with statin therapy improves patency to one year [17, 18]. Beyond this, no substantive impact has been made in preventing the development of vein graft atherosclerosis or improving patency. Patency of the SVG to one year is also less in smaller vessels, younger age group patients and grafts to the circumflex and right coronary systems and to those vessels with poor runoff [10, 19]. No medical therapeutic or endothelial antibody trial has improve patency beyond one year, thus the concept of external vein support is appealing [20–22].

Saphenous vein graft patency at one year has been reported to be as low as 75% and at ten years 50% [11, 18] over the subsequent 10 years there is a graft loss of 1-2% per year due to the development of vein graft atherosclerotic changes [10]. The eSVS Mesh was developed to address the lower patency saphenous vein. The mesh graft supports and downsizes the SVG by up to 35% and prevents over expansion of the saphenous vein to no more than 8% of its luminal diameter thus preventing endothelial injury during acute exposure to arterial pressure. Animal experiments have documented arterial like healing of the supported grafts as compared to unsupported grafts [13, 14]. Early graft patency is dependent on the quality of the vein harvested, diameter of the coronary artery to which it is grafted, blood flow through the graft and ejection fraction. Thus, early patency may not be impacted by the eSVS Mesh as the device is designed to prevent endothelial injury and vein over distention, thus obviating the development of graft atherosclerosis over time. Genoni found that early graft patency is not negatively affected by use of the eSVS Mesh, important data when using newly implantable foreign material, assuring patient safety from potential device related complications [23].

Surgical technique can be applied using those methods of any individual surgeon: Running versus interrupted suture; proximal versus distal first; on pump or off pump revascularization. Most importantly during construction of the anastomosis, a cobra head must be created so the graft is angulated properly both at the proximal and distal end [16]. Transit-Time Doppler Flow measurement is recommended for intra-operative assessment of patency as the external support mesh does not interfere with Doppler signals [24]. Intermediate term patency data is, however, limited.

A first in man pivotal study was performed as an international multi-center randomized trial with patients receiving grafts randomized to the right and left system thus each patient served as their own control (personal communication). This study showed equivalency in patency between the supported and unsupported grafts. However, several lessons were learned from this study which may further improve the patency of supported grafts.

First, the smallest size, a 3 mm mesh was eliminated as closure rate was increased in this group and secondly, the need for an incision in the vein graft to create a cobra-head anastomosis in completion of both the proximal and the distal anastomosis is necessary to assure the each graft lies properly without kinking. Creating a cobra head for proper lie of the grafts is particularly important as those grafts constructed without this technical step had poor intermediate results [25]. Finally, accurate measurement of the vein diameter and double wall thickness were important in selecting appropriate veins to support.

## Conclusion

The current study demonstrated excellent patency at 7.2 +/- 3.7 months of 92%. While there were no controls and this post-market study was not randomized, compared to the literature objective performance criteria, graft patency at this time frame is improved [18, 19]. The eSVS Mesh thus does not negatively affect early graft patency, is adaptable to varying surgical techniques and shows improvement in graft patency at the intermediate term. Further prospective randomized trials are warranted and longer term follow-up is necessary. Due to graft support and improved flow characteristics, the development of vein graft disease should be retarded and a greater impact on long term as opposed to short term patency expected. Studies are ongoing.

## Authors’ original submitted files for images

Below are the links to the authors’ original submitted files for images. Authors’ original file for figure 1 Authors’ original file for figure 2

## Abbreviations

AHD: American Hospital Dubai
CABG: Coronary Artery Bypass Grafting
CTA: Computerized tomographic angiography
FDA: Food and drug administration
SVG: Saphenous vein graft.
