# Supplementary figures and images for: Computerized tomographic angiography in patients having eSVS Mesh® supported coronary saphenous vein grafts: intermediate term results
Source: J Cardiothorac Surg. 2014 Aug 13;9:126. doi: 10.1186/1749-8090-9-126 (PMC4413552; doi:10.1186/1749-8090-9-126)

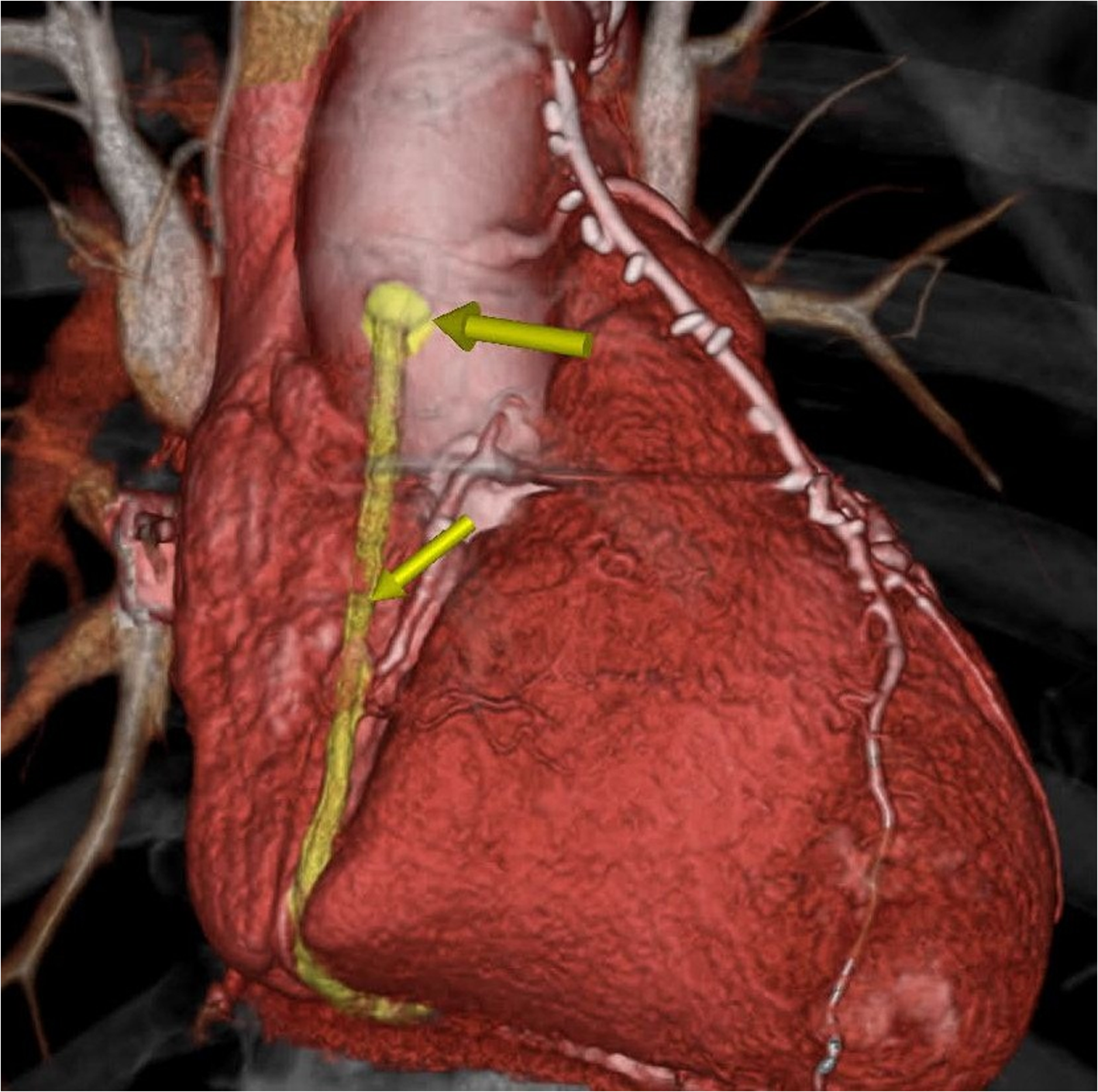

Supplement: Supplementary file 1 — Authors’ original file for figure 1 [file 13019_2013_1575_MOESM1_ESM.tif]

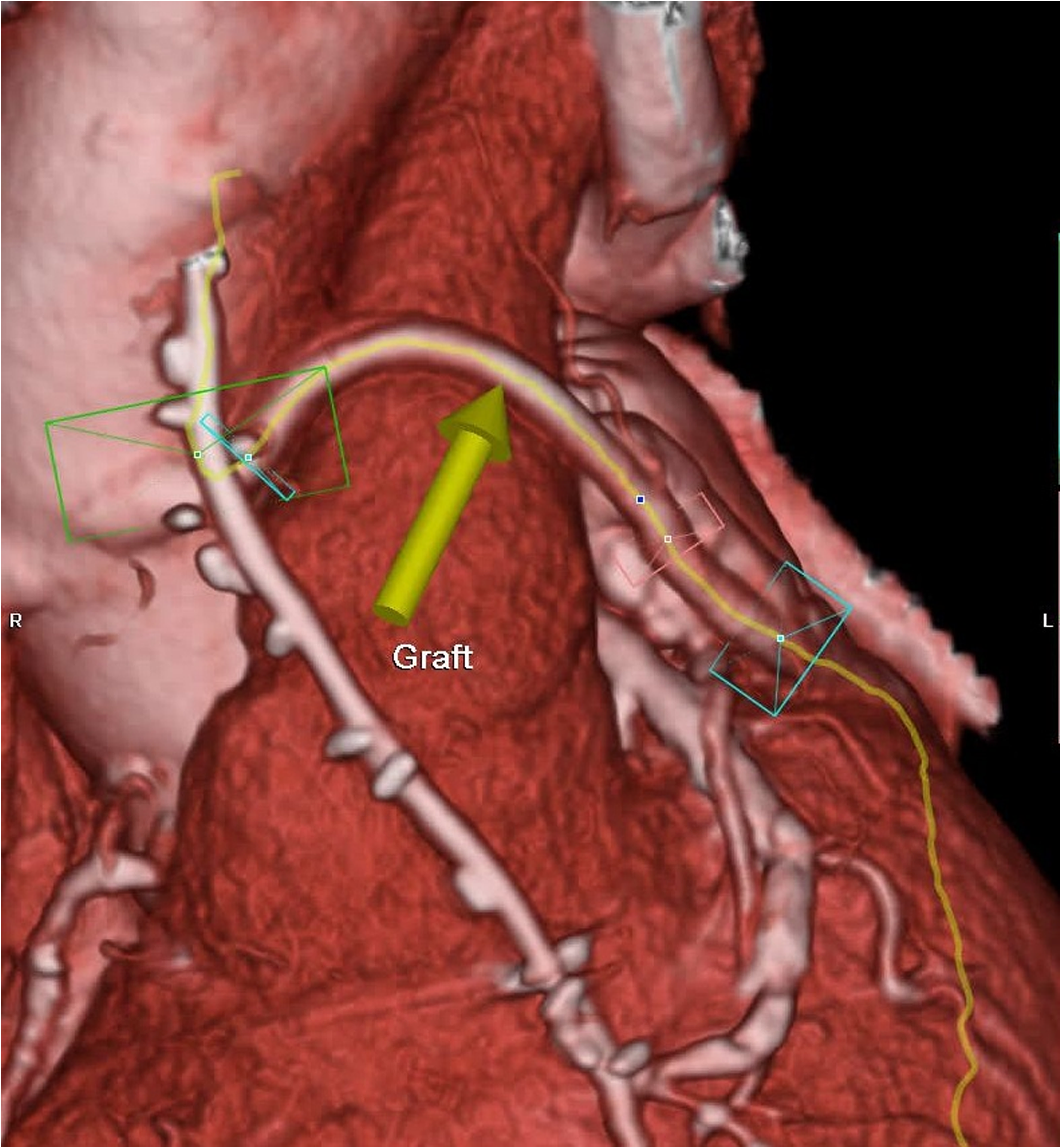

Supplement: Supplementary file 2 — Authors’ original file for figure 2 [file 13019_2013_1575_MOESM2_ESM.tif]
